# Whole-Genome Sequence of a Reassortant H5N6 Avian Influenza Virus Isolated from a Live Poultry Market in China, 2013

**DOI:** 10.1128/genomeA.00706-14

**Published:** 2014-09-11

**Authors:** Xian Qi, Lunbiao Cui, Huiyan Yu, Yiyue Ge, Fengyang Tang

**Affiliations:** Key Lab of Enteric Pathogenic Microbiology (Ministry of Health), Jiangsu Provincial Center for Disease Prevention and Control, Nanjing, China

## Abstract

An avian influenza virus, A/environment/Zhenjiang/C13/2013(H5N6), was isolated from a live poultry market in eastern China. Phylogenetic analysis showed that the isolate was a novel reassortant virus with a neuraminidase (NA) gene from H6N6 viruses and the other seven genes from H5N1 viruses, which may pose a potential threat to human and animal health.

## GENOME ANNOUNCEMENT

Since the late 1990s, multiple genotype H5N1, H9N2, and H6N6 influenza viruses have been circulating in poultry populations in China (1–3), which provide abundant gene pools for further inter-or-intro subtype reassortment. In May 2014, a fatal human infection with H5N6 avian influenza virus (AIV) was confirmed in China (China CDC, http://www.chinacdc.cn/mtdx/crbxx/201405/t20140512_96874.htm). However, the epidemiology and biological characterization of the H5N6 virus are still unknown in China.

Live poultry markets may play an important role in the evolution and transmission of avian influenza viruses (4). In December 2013, an H5N6 AIV strain, A/environment/Zhenjiang/C13/2013(H5N6) (en/c13), was isolated from sewage in a live poultry market where ducks were sold in Jiangsu, China. The eight genes were sequenced by MiSeq (Illumina). Sequences were assembled by CLC Genomics workbench (CLC Bio).

The full lengths of the polymerase PB2 and PB1, polymerase acidic protein (PA), hemagglutinin (HA), nucleoprotein (NP), neuraminidase (NA), matrix (M), and nonstructural (NS) genes were 2,341, 2,341, 2,233, 1,776, 1,565, 1,464, 1,027, and 890 nucleotides, respectively. Phylogenetic analyses showed that the NA gene belonged to the same clade with H6N6 viruses currently circulating in China, such as A/duck/Guangxi/Gxd-7/2011 t (up to 98% nucleotide identity with the reference strains), whereas the other seven genes were found to be more similar to those of eastern Asian H5N1 AIV strains (95 to 99% nucleotide identity).

The amino acid sequence of the cleavage site in the HA protein was PLREKRRKR↓GLF, with characteristics of high-pathogenicity AIVs (5). The receptor binding site in the viral HA gene possesses the residues Gln 226 and Gly 228 (H3 numbering), indicating an avian-like receptor binding specificity. The amino acids at residues 591, 627, and 701 in the PB2 protein were Gln, Glu, and Asp, respectively, a characteristic of avian replication preference. There is an “avian-like” ESEV motif at the NS1 C terminus, which may increase the virulence of H5 viruses in mammals.

In conclusion, the en/c13 strain may be a novel natural reassortant with the NA gene from H6N6 and the other seven genes from H5N1 AIVs, which may pose a potential threat to human and animal health. This study also highlights the importance of surveilling the evolution of AVIs in live poultry markets.

### Nucleotide sequence accession numbers.

The genome sequences of A/environment/Zhenjiang/C13/2013(H5N6) have been deposited in GenBank under accession numbers KJ938655 to KJ938662.
